# Causal effects of gut microbiota on the risk of periodontitis: a two-sample Mendelian randomization study

**DOI:** 10.3389/fcimb.2023.1160993

**Published:** 2023-05-25

**Authors:** Shulu Luo, Weiran Li, Qianqian Li, Mengqi Zhang, Xun Wang, Shuyi Wu, Yan Li

**Affiliations:** Department of Prosthodontics, Hospital of Stomatology, Guangdong Provincial Key Laboratory of Stomatology, Guanghua School of Stomatology, Sun Yat-sen University, Guangzhou, Guangdong, China

**Keywords:** periodontitis, gut microbiota, Mendelian randomization, causal effect, risk factor

## Abstract

**Introduction:**

The oral cavity and the gut tract are interconnected, and both contain abundant natural microbiota. Gut microbiota may interact with oral flora and participate in the development of periodontitis. However, the specific role of certain gut microbiota taxa for periodontitis has not been investigated. Mendelian Randomization is an ideal method to explore causal relationships avoiding reverse causality and potential confounding factors. Thus, we conducted a two-sample Mendelian Randomization study to comprehensively reveal the potential genetic causal effect of gut microbiota on periodontitis.

**Methods:**

SNPs strongly associated with 196 gut microbiota taxa (18,340 individuals) were selected as instrument variables, and periodontitis (17,353 periodontitis cases and 28,210 controls) was used as the outcome. The causal effect was analyzed via random effect inverse variance-weighted, weighted median, and MR-Egger. The sensitivity analyses were conducted using Cochran’s Q tests, funnel plots, leave-one-out analyses, and MR-Egger intercept tests.

**Results:**

Nine gut microbiota taxa (*Prevotella* 7, *Lachnospiraceae* UCG-008, *Enterobacteriales*, *Pasteurellales*, *Enterobacteriaceae*, *Pasteurellaceae*, *Bacteroidales* S24.7 group, *Alistipes*, and *Eisenbergiella*) are predicted to play a causal role in enhancing the risk of periodontitis (*p*< 0.05). Besides, two gut microbiota taxa (*Butyricicoccus* and *Ruminiclostridium* 6) have potentially inhibitive causal effects on the risk of periodontitis (*p*< 0.05). No significant estimation of heterogeneity or pleiotropy is detected.

**Conclusion:**

Our study demonstrates the genetic causal effect of 196 gut microbiota taxa on periodontitis and provides guidance for the clinical intervention of periodontitis.

## Introduction

As the leading cause of missing teeth and the most prevalent oral disease, periodontitis is a chronic, irreversible, and destructive inflammatory disease that damages the soft tissue and alveolar bone supporting the teeth (Papapanou et al., 2018). Currently, periodontitis has imposed an unbearable burden on humans. For individuals, periodontitis tends to have a tremendous impact on the patient’s masticatory function, aesthetics, and psychology, which cause a significant reduction in their life quality (Barbe et al., 2020). Periodontitis is also associated with serious systematic health problems, such as cardiovascular diseases, chronic lower respiratory disorders, and diabetes (Chapple et al., 2013; Tonetti et al., 2013; Kelly and El Karim, 2020). Globally, periodontitis is estimated to affect around 50% of adults in its mild state and is recorded as the sixth most prevalent disease in its severe state (Genco and Sanz, 2020). Furthermore, the prevalence of periodontitis increases with age and the economic impact of periodontitis accounts for a significant portion of the annual global economic burden of dental disease (Billings et al., 2018). Since periodontitis’ socioeconomic and healthcare impacts are enormous, it’s urgent to identify risk factors for periodontitis to prevent its occurrence. However, implementing public health models to prevent periodontitis is insufficient (Janakiram and Dye, 2020). Existing studies have demonstrated that many risk factors are associated with periodontitis, including smoking, alcohol consumption, poor oral hygiene, systematic health, and so on (Bartold, 2018; Baumeister et al., 2021). To further reduce the periodontitis burden, more emphasis should be laid on extra potentially modifiable risk factors.

Physiologically, the oral cavity and the gut are continuous areas connected by the gastrointestinal tract in the digestive system, and both are abundant in microorganisms. Periodontitis is triggered by dysregulated intraoral microbial communities and aberrant immune responses (Hajishengallis et al., 2020). Meanwhile, the gut microbiota is demonstrated to involve in various physiological regulations and the progression of many diseases, such as cardiovascular diseases, autoimmune diseases, and tumors (Zhou et al., 2019; Xu et al., 2022). It is of concern whether gut microbiota, as an extra-oral but oral-connected microbial community, can cause or mediate periodontitis. Traditionally, the gut microbiota was not considered to be one of the risk factors for periodontitis. Over the past few years, there has been increasing evidence of an association between gut microbiota and periodontitis. Some experimental studies point out that gut microbiota can modulate bone metabolism and play an essential role in regulating periodontal bone remodeling (Yan et al., 2016), which can affect the development of periodontitis (Jia et al., 2019). Gut microbiota may also mediate the impact of periodontitis on systemic diseases including prediabetes (Li et al., 2021). However, existing studies have not explored the causal relationship between gut microbiota and periodontitis or focused on the exact role of specific gut microbiota taxa on periodontitis. Given the above findings, we hypothesize that variations in the gut microbiota composition contribute to periodontitis. However, both gut microbiota composition and periodontitis share several risk factors including diets, smoking, alcohol consumption, and stress, which might cause spurious correlations as confounders (Bjorkhaug et al., 2019; Baumeister et al., 2021). Therefore, distinct populations studied, reverse causality, and potential confounding factors in current studies hinder the inference of the causal effect between gut microbiota and periodontitis. What’s more, the gut microbiota is a complex microorganism community containing diverse taxa, and research on the function of specific gut microbiota taxa for periodontitis is absent. Overall, confirmation of a causal relationship for this correlation and which microbiota taxa are most relevant is essential for clinical practice in the management of periodontitis.

Mendelian randomization (MR) is an alternative method to interpret observational bias, using genetic variation normally single nucleotide polymorphisms (SNPs) as instrumental variables (IV), to detect causal relationships between exposure and disease outcomes (Yavorska and Burgess, 2017). If the exposure is causally associated with the outcome, the IV related to the exposure will proportionally affect the outcome (Chen et al., 2021). The MR studies are similar to randomized controlled trials (RCT) because the probability is the same in the inheritance of either allele to an individual at random (Emdin et al., 2017). Since genetic variants are usually not associated with confounding factors, MR is more powerful in avoiding reverse causal associations and confounding factors than traditional observational studies (Hu et al., 2022). Previously, many MR studies have been applied to elucidate modest risk factors for various diseases, including cancers, cardiovascular diseases, and so on, which is effective in solving problems in epidemiology (Higbee et al., 2021). In addition, Dmitry. S reports genome-wide association studies (GWAS) for periodontitis in 17,353 participants (Shungin et al., 2019), which enables the conduction of an MR study on the potential causal links between gut microbiota and periodontitis, minimizing population differences, reverse causation, and confounding factors that interfere with the analyses.

Herein, we performed a two-sample MR study based on public large-scale GWAS data of gut microbiome and periodontitis to reveal the possible causal effects of 196 gut microbiota taxa on periodontitis. Eventually, we confirm the role of specific gut microbiota taxa in increasing or reducing the risk of periodontitis, most of which haven’t been involved in the fields of stomatology. Our findings thus not only expand the taxa of gut microbes associated with periodontitis but also reveal their specific causal relationship with periodontitis, providing a new strategy for the clinical control of periodontitis.

## Materials and methods

### Study design

**Figure 1:**
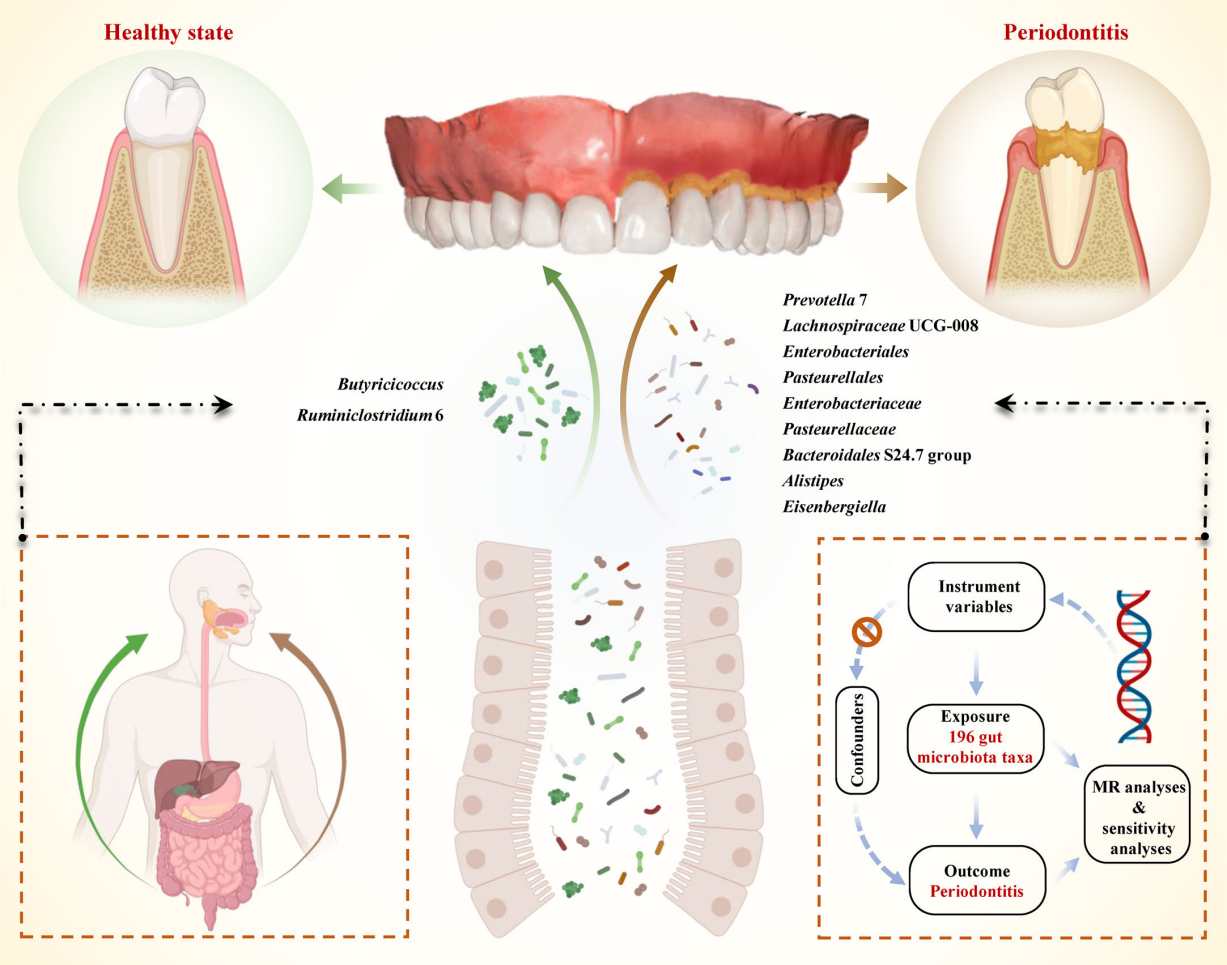
Schematic illustration of the causal relationship between gut microbiota and periodontitis through MR analyses. An overview of the two-sample MR study revealed the causal effect of specific gut microbiota taxa on periodontitis. There existed 9 taxa of gut microbiota that accelerated the initiation of periodontitis and 2 taxa of gut microbiota that reduced the risk of periodontitis. (MR, Mendelian randomization; SNPs, single nucleotide polymorphisms).

In this study, we performed comprehensive MR analyses to reveal the causal effect between 196 gut microbiota taxa and periodontitis. The framework of our study design is presented in **Figure 1**. The exposure of interest was 196 gut microbiota taxa, and the outcome was periodontitis. The instrument variables for the exposure and corresponding information in the outcome were obtained and harmonized. Then MR analysis - included three approaches were performed and sensitivities analyses were conducted. A well-designed MR should follow three assumptions: (i) the genetics variant was strongly correlated with the exposure of interest (the gut microbiota); (ii) the genetics variant was not correlated with potential risk factors of the outcome (periodontitis) (*p*< 1×10^-5^); (iii) the genetic variant played role in the outcome only through exposure (Emdin et al., 2017). All these hypotheses were well-handled in our analyses. For the first hypothesis, we extracted strong instrument variables and calculated their F statistics to evaluate their strength. Since SNPs on each chromosome were randomly assigned during meiosis according to Mendel’s second law, the second hypothesis actually has been met from the study design (Veller et al., 2019). In addition, we search the PhenoScanner to remove SNPs associated with confoundings (Staley et al., 2016; Kamat et al., 2019). For the third hypothesis, we evaluated the pleiotropy using MR-Egger intercept analysis.

### Data sources and instrumental variable selections

Previously, the MiBioGen consortium analyzed genome-wide genotypes and fecal microbiome data from 18,340 individuals mostly from North America, Israel, South Korea, and European (Kurilshikov et al., 2021). They performed strict quality control and those left for analysis should have a pointwise imputation quality control > 0.4. Taxa with prevalence low than 20% was discarded. The microbiome GWAS was adjusted by age, sex, technical covariates, and genetic principal components. The cutoff thresholds included: minor allele frequency > 0.05, and SNP-wise call filtering > 0.95. Alexander Kurilshikov and his colleagues reported the significant loci of host genetic variation in relation to microbial taxa (Kurilshikov et al., 2021). After excluding 15 unknown taxa, there were 196 known bacterial taxa, including 9 phyla, 16 classes, 20 orders, 32 families, and 119 genera (**Table S1**). The criteria for the 196 taxa included: (i) *p<* 1×10^-5^, since limited SNPs could be obtained under the genome-wide significance (*p*< 5×10^-8^) and such relaxed threshold have also been applied in many studies (Sanna et al., 2019); (ii) Linkage disequilibrium (LD) test was performed to remove linked SNPs r^2^< 0.1 within a window of 500 kilobase pair (Ni et al., 2022); (iii) The F-statistic was calculated for each SNP and SNPs with F< 10 were eliminated to avoid weak instruments bias (Burgess et al., 2011). The F-statistic for each SNP was calculated with a formula as


$$\text{F = }\frac{\text{R}2\;}{\left(1-\text{R}2\;\right)}\text{× (N-2)}$$

In the formula, R^2^ means variance of exposure explained by instrument variable; N indicates sample size. And the variance of exposure explained by the instrument variable was calculated with a formula as


$$\text{R}2\;=\frac{\beta 2}{\left(\beta 2+\text{se}2\;\times \text{N}\right)}$$

In this formula, β indicated effect size for the genetic variant of interest; se indicated a standard error for β; N indicated sample size.

After strict selections, the identified IVs and corresponding F statistics were presented in **Table S2**.

The GWAS of periodontitis was from the GLIDE consortium which included 17,353 periodontitis cases and 28,210 controls (Shungin et al., 2019). Participants were of European ancestry. Periodontitis cases were categorized in compliance with the Community Periodontal Index (World Health Organization, 2013) or the Centers for Disease Control and Prevention/American Academy of Periodontology case definition in terms of the probing depths and the amount of deep periodontal pockets (Page and Eke, 2007). The SNP information of instrument SNPs in outcomes was obtained and harmonized with that of the exposure.

### Mendelian randomization and sensitivity analyses

The MR and sensitivity analysis methods were consistent with published studies (Chen et al., 2021; Chen X. et al., 2022). Random effect inverse variance-weighted (IVW) was used as the main analysis for it was the most robust analysis and could provide a modest estimate with the presence of heterogeneity. Besides, we performed the weighted median (WM) and MR-Egger to validate the robustness of IVW estimates. MR-Egger regression could provide a test for unbalanced pleiotropy and considerable heterogeneity. When pleiotropy exists, the MR-Egger estimates were more convincing than IVW estimates (Bowden et al., 2015). And when at least half of the weighted variance provided by horizontal pleiotropy was valid, the WM estimates could provide robust estimates of effect (Bowden et al., 2016). In short, a significant estimate provided by IVW with the same direction of estimates provided by WM and MR-Egger was treated as a significant estimate.

We also conducted a series of sensitivity analyses, including Cochran’s Q tests, funnel plots, leave-one-out analyses, and MR-Egger intercept tests. To be specific, heterogeneity was detected by Cochran’s Q tests. The intercept term derived from MR-Egger regression was utilized to evaluate pleiotropy. The leave-one-out analyses were conducted to determine whether the causal estimate was driven by any single SNP.

All analyses were performed with the “Two Sample MR” (version 0.5.6) package in R software (version 4.2.1). A two-sided *p*< 0.05 was set as significant. All estimations were expressed as odds ratios (OR) per standard deviation (SD) increment of the corresponding exposure.

## Results

### An overview of IVs in taxa

Through screening the genome-wide significance threshold (*p*< 1×10^-5^), LD tests, harmonizing, and verifying *F*-statistics, each of the 196 bacterial taxa gets multiple SNPs as their IVs. The *F-*statistics of all retained SNPs are over 10, indicating sufficient correlation strength between IVs and corresponding bacterial taxon. Thus, our study has no weak instrument bias.

### Associations of gut bacterial taxa with periodontitis

**Figure 2:**
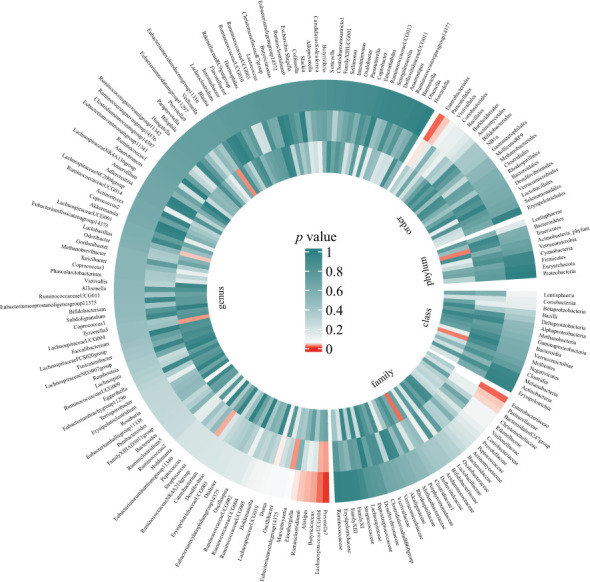
Preliminary MR analyses for the associations between gut microbiota and the risk of periodontitis. The circle from the outer to the inner represented the IVW, WM, and MR-Egger estimates, respectively. Gut microbiota was classified in order, phylum, class, family, and genus. The shades of color were reflections of the magnitude of the *p*-value as the label inside the circle. (MR, Mendelian randomization; IVW, inverse variance-weighted; WM, weighted median).

**Figure 3:**
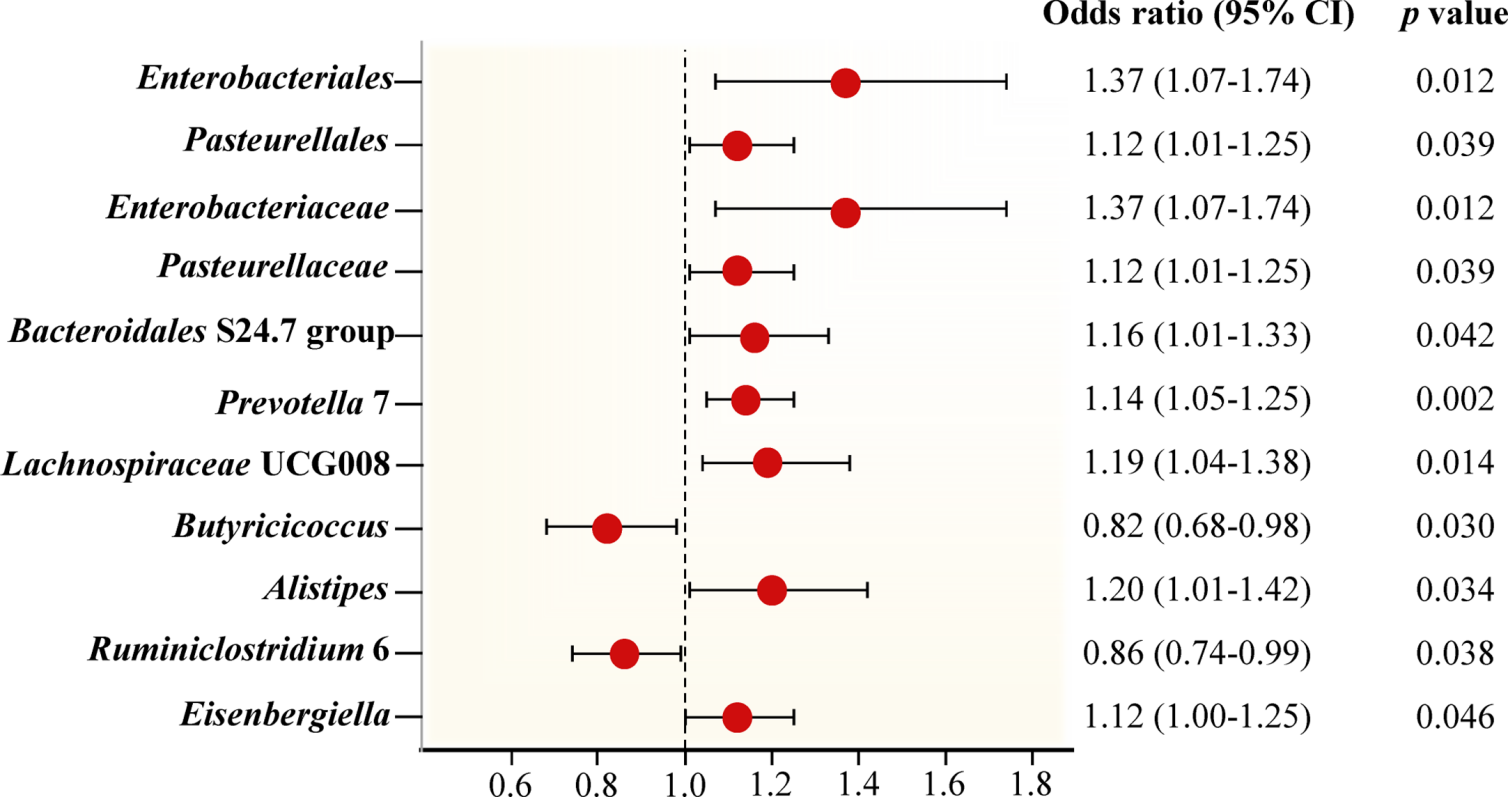
Forest plot of Mendelian randomization estimates between Gut microbiota and periodontitis. The figure showed the IVW estimates of significantly periodontitis-associated gut microbiota taxa. The red dots represent the IVW estimates, and the black bars represent the 95% confidence intervals of IVW estimates. The OR > 1 indicates increased risk while< 1 indicates decreased risk.

Preliminary results for the analyses of associations between genetically proxied gut bacterial taxa and risks of periodontitis are presented in **Figure 2**; **Table S3**. Among the 196 bacterial taxa, we find 11 gut microbiota taxa causally associated with periodontitis (**Figure 3**). The position of instrumental SNPs of the causal microbiota taxa and their nearest genes were listed in **Table S4**.

**Figure 4:**
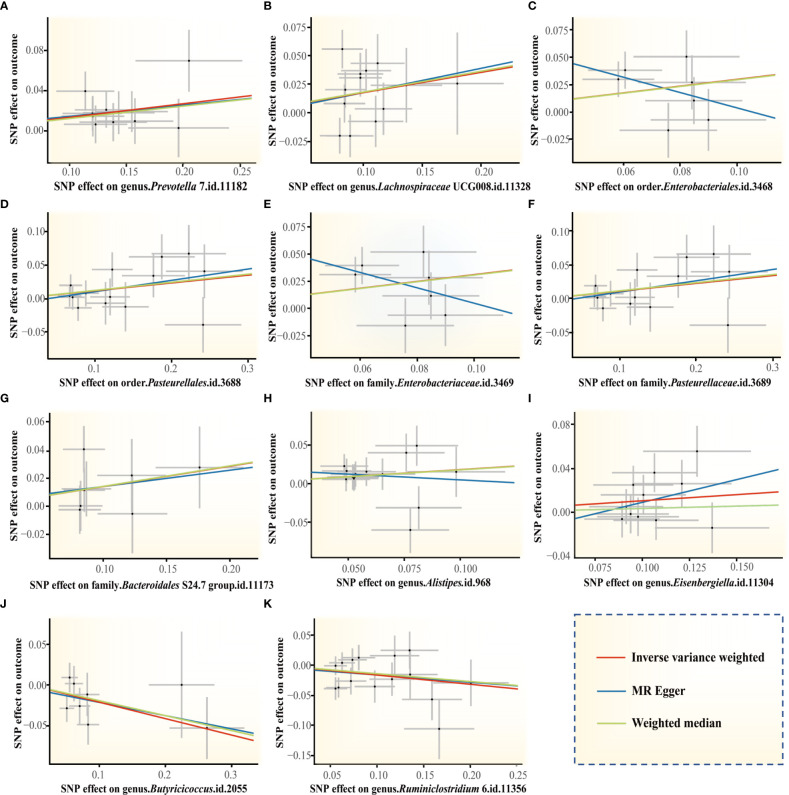
Scatter plots of the MR estimates for the significant causality of 11 gut microbiota taxa and the risk of periodontitis. **(A)** The causal effect of the genus *Prevotella* 7 on periodontitis; **(B)** The causal effect of genus *Lachnospiraceae* UCG008 on periodontitis; **(C–K)** Potential causal effect of 9 other gut microbiota taxa on periodontitis. The lines implying positive correlations moved diagonally upward from left to right, indicating a facilitative effect of gut microbiota on periodontitis. The horizontal and vertical lines indicated each correlation’s 95% confidence interval. The lines implying negative correlations move diagonally downward from left to right, indicating the inhibitory effect of gut microbiota on periodontitis. (MR, Mendelian randomization; SNPs, single nucleotide polymorphisms).

The genus *Prevotella* 7 is found to be positively associated with periodontitis, suggesting that genus *Prevotella* 7 in the human gut is causally related to an increased risk of periodontitis (IVW OR = 1.14, 95% confidence interval (CI)1.05-1.25, *p* = 0.002). The above result is furtherly confirmed by WM analyses (OR = 1.13, 95% CI 1.01-1.26, *p* = 0.028). The causal assessment from the MR-Egger analysis also supports consistent correlation but is not significant (OR = 1.12, 95% CI 0.67-1.88, *p* = 0.681) (**Figure 4A**; **Table 1**). The genus *Lachnospiraceae* UCG008 also has a progressive effect on periodontitis (IVW OR = 1.19, 95% CI 1.04-1.38, *p* = 0.014). The WM analysis shows similar results (OR = 1.20, 95% CI 1.02-1.42, *p* = 0.032). However, the MR-Egger analysis still exhibits consistent but insignificant trends (OR = 1.24, 95% CI 0.53-2.89, *p* = 0.633) (**Figure 4B**; **Table 1**).

**Table 1:** MR estimates for the relationship between genetically instrumented gut microbiota and periodontitis.

Exposure	Method	OR	95% CI	*p*-value
*Enterobacteriales*	IVW	1.37	1.07-1.74	0.012
	WM	1.36	1.01-1.85	0.046
	MR-Egger	0.49	0.13-1.81	0.336
*Pasteurellales*	IVW	1.12	1.01-1.25	0.039
	WM	1.13	0.96-1.32	0.130
	MR-Egger	1.18	0.92 -1.53	0.2163
*Enterobacteriaceae*	IVW	1.37	1.07-1.74	0.012
	WM	1.36	1.00-1.86	0.051
	MR-Egger	0.49	0.13-1.81	0.336
*Pasteurellaceae*	IVW	1.12	1.01-1.25	0.039
	WM	1.13	0.97-1.31	0.117
	MR-Egger	1.18	0.92-1.53	0.216
*Bacteroidales* S24.7 group	IVW	1.16	1.01-1.33	0.042
	WM	1.16	0.97-1.38	0.113
	MR-Egger	1.12	0.64-1.97	0.702
*Prevotella* 7	IVW	1.14	1.05-1.25	0.002
	WM	1.13	1.01-1.26	0.028
	MR-Egger	1.12	0.67-1.88	0.681
*Lachnospiraceae* UCG008	IVW	1.19	1.04-1.38	0.014
	WM	1.20	1.02-1.42	0.032
	MR-Egger	1.24	0.53-2.89	0.633
*Butyricicoccus*	IVW	0.82	0.68-0.98	0.030
	WM	0.83	0.64-1.08	0.161
	MR-Egger	0.85	0.60-1.20	0.384
*Alistipes*	IVW	1.20	1.01-1.42	0.034
	WM	1.20	0.96-1.50	0.117
	MR-Egger	0.86	0.37-2.00	0.736
*Ruminiclostridium* 6	IVW	0.86	0.74-0.99	0.038
	WM	0.88	0.72-1.08	0.211
	MR-Egger	0.89	0.60-1.30	0.548
*Eisenbergiella*	IVW	1.12	1.00-1.25	0.046
	WM	1.04	0.90-1.21	0.580
	MR-Egger	1.52	0.63-3.63	0.372

MR, Mendelian randomization; CI, confidence interval; OR, odds ratio; IVW, inverse variance-weighted; WM, weighted median.

In addition, we identify potential causal relationships between the other 9 taxa and periodontitis, as the IVW analyses results for all 9 phenotypes show significant differences (*p*< 0.05, **Table 1**). Among them, 7 taxa are determined to have a potential positive causal effect on periodontitis to increase the risk of periodontitis (**Figures 4C–I**), specifically containing the order *Enterobacteriales* (IVW OR = 1.37, 95% CI 1.07-1.74, *p* = 0.012), the order *Pasteurellales* (IVW OR = 1.12, 95% CI 1.01-1.25, *p* = 0.039), the family *Enterobacteriaceae* (IVW OR = 1.37, 95% CI 1.07-1.74, *p* = 0.012), the family *Pasteurellaceae* (IVW OR = 1.12, 95% CI 1.01-1.25, *p* = 0.039), the family *Bacteroidales* S24.7 group (IVW OR = 1.16, 95% CI 1.01-1.33, *p* = 0.042), the genus *Alistipes* (IVW OR = 1.20, 95% CI 1.01-1.42, *p* = 0.034), and the genus *Eisenbergiella* (IVW OR = 1.12, 95% CI 1.00-1.25, *p* = 0.046). Consequently, the above 7 taxa are confirmed to causally increase the risk of periodontitis. On the contrary, 2 taxa including the genus *Butyricicoccus* (IVW OR = 0.82, 95% CI 0.68-0.98, *p* = 0.030) and the genus *Ruminiclostridium* 6 (IVW OR = 0.86, 95% CI 0.74-0.99, *p* = 0.038) are identified as having a negative causal effect on periodontitis (**Figures 4J, K**) and tending to causally reduce the risk of periodontitis. Except the order *Enterobacteriales*, the family *Enterobacteriaceae*, and the genus *Alistipes*, the other MR analyses (WM and MR-Egger) present consistent results with the corresponding IVW analyses for the remaining 6 taxa.

For the order *Enterobacteriales*, the family *Enterobacteriaceae*, and the genus *Alistipes*, the causal correlations estimated by MR-Egger are the reverse of that detected by the other two MR analyses, although not significant. Therefore, the interpretation of these potential causal effects should be cautious. More investigations are needed. Nevertheless, due to the absence of horizontal pleiotropy and heterogeneity (*p* > 0.05, explained below), the causal relationships obtained by IVW are more accurate than the results from MR-Egger (Bowden et al., 2016; Chen et al., 2021; Chen M. et al., 2022). Consequently, it’s reasonable to recognize the IVW estimates for the order *Enterobacteriales*, the family *Enterobacteriaceae*, and the genus *Alistipes*, that is, they may increase the risk of periodontitis.

### Sensitivity analyses and detection of pleiotropy

To avoid excessive bias effects, pleiotropic analyses are conducted. The pleiotropies are absent in the IVs of the mentioned 11 taxa causally associated with periodontitis (*p* > 0.05) and the conclusions are supported by the leave–one–out sensitivity (**Figure S1**). Funnel plots indicate that causal associations are less likely to be influenced by potential biases with SNPs symmetrically distributed (**Figure S2**). In view of heterogeneous results, Cochran’s Q tests demonstrate no evidence of heterogeneity among 11 taxa (*p* > 0.05). Besides, MR-Egger intercept tests exhibit no indication of horizontal pleiotropy within 11 taxa (**Table 2**). In summary, our MR analyses are verified to be reliable and robust. All these results suggest that the identified causal relationships between gut microbiota and periodontitis are likely to be mediated by the above gut bacterial taxa.

**Table 2:** MR-Egger test for directional pleiotropy and heterogeneity.

Exposure	Intercept	*p*-value	Q	Q_ *p*-value
*Enterobacteriales*	0.004	0.829	17.518	0.420
*Pasteurellales*	0.036	0.173	8.640	0.733
*Enterobacteriaceae*	-0.003	0.937	5.308	0.380
*Pasteurellaceae*	0.000	0.983	10.971	0.531
*Bacteroidales* S24.7 group	0.017	0.646	8.052	0.234
*Prevotella* 7	0.003	0.779	8.595	0.968
*Lachnospiraceae* UCG008	-0.002	0.880	7.863	0.852
*Butyricicoccus*	0.000	0.984	13.225	0.279
*Alistipes*	0.025	0.528	16.175	0.135
*Ruminiclostridium* 6	-0.019	0.361	17.878	0.397
*Eisenbergiella*	0.001	0.985	2.532	0.865

MR, Mendelian randomization; Q, heterogeneity statistic Q.

## Discussion

In our research, a two-sample MR study successfully determines that partial gut microbiota can facilitate or prevent periodontitis. The results are examined by several analyses, namely IVW, WM, and MR-Egger analyses, almost all of which show consistent causal associations. The genus *Prevotella* 7, the genus *Lachnospiraceae* UCG-008, the order *Enterobacteriales*, the order *Pasteurellales*, the family *Enterobacteriaceae*, the family *Pasteurellaceae*, the family *Bacteroidales* S24.7 group, the genus *Alistipes*, and the genus *Eisenbergiella* play causal roles in promoting the initiation of the periodontitis, while the genus *Butyricicoccus* and the genus *Ruminiclostridium* 6 causally reducing the risk of periodontitis. Our findings fill the knowledge gap of whether gut microbiome can contribute to periodontitis and which taxa can accelerate or inhibit the initiation of periodontitis.

*Prevotella* is identified to activate key pathogenic species of periodontitis especially *P. gingivalis* that can result in periodontal diseases at low abundance (Hajishengallis et al., 2011). The genome of *Prevotella* is highly plastic and diverse, which favors its resistance to multiple exogenous factors, adaptation to variable environments, and generation of virulence (Purushe et al., 2010). This characteristic may account for the association between *Prevotella* and several oral infectious diseases. According to observational experiments, the genus *Prevotella* 7 in the gut of patients with periodontal diseases is significantly increased compared with that of populations without periodontal diseases (Abusleme et al., 2021). In our study, a higher abundance of the genus *Prevotella* 7 indicates a higher risk of periodontitis, suggesting the genus *Prevotella* 7 detected in fecal samples can be considered as a predictive biomarker and a target for efficient intervention in periodontitis.

To be noted, we found novel positively related taxa that haven’t been reported in previous literature. Among them, the genus *Lachnospiraceae* UCG-008 is positively correlated to the release of inflammatory factors such as IL-6, HS-CRP, and TNF-α, indicating that reducing the numbers of *Lachnospiraceae* UCG-008 is beneficial for controlling inflammation and can function in controlling periodontitis (Zhu et al., 2020). The pathogenic members of the order *Pasteurellales* and the family *Pasteurellaceae* are partially distributed in the mucosal of the oral cavity (Christensen et al., 2020). Our Mendelian study has shown that these two taxa distributed in the gut increase the risk of periodontitis. Combining previous studies and our findings, we assume that the order *Pasteurellales* and the family *Pasteurellaceae* may deteriorate periodontitis by regulating their abundance and pathogenicity in the oral cavity. The family *Bacteroidales* S24.7 group, one of the major components of gut microbiota, can trigger infections by stimulating immunity and synthesizing virulence factors (Ormerod et al., 2016). However, an observational study presents contradictory results showing the anti-inflammatory effects of metabolites of the family *Bacteroidales* S24.7 group. The inconsistency between studies may be ascribed to the highly individual variations in gut microbiological composition and the complicated multifactorial properties of inflammatory diseases (Eckburg et al., 2005). Our MR study can evade these issues by providing validation from a genetic perspective, obtaining clear evidence that the family *Bacteroidales* S24.7 group promotes the risk of periodontitis. Next, according to previous observational studies, the increase in the genus *Eisenbergiella* is associated with the deterioration of some chronic diseases, particularly pro-inflammatory illnesses which may include periodontitis (Bailen et al., 2020).

In addition to the above 9 taxa promoting periodontitis, we also indicate 2 negatively causative gut microbiota taxa (the genus *Butyricicoccus* and the genus *Ruminiclostridium* 6) that were first reported to be associated with periodontitis. Both the genus *Butyricicoccus* and the genus *Ruminiclostridium* 6 can degrade polysaccharides through autocrine multienzyme complexes to produce short-chain fatty acids such as butyrate, which acts as a valid anti-inflammatory mediator (Devriese et al., 2017; Wang et al., 2018). The biological function of butyrate is to maintain the intestinal epithelial barrier, balance gut microbiota, inhibit the expression of destructive cytokine, and regulate immunity and inflammation (Xiao et al., 2020), which may be the mechanisms of these two taxa to reduce risks of periodontitis. Combined with the existing results, our MR study suggests that we can possibly achieve prevention and control of periodontitis by increasing the abundance of the genus *Butyricicoccus* and the genus *Ruminiclostridium* 6 in various ways. Taken together, the correlation between mentioned gut microbiota taxa and periodontitis as verified by our MR study is reasonable.

Since the oral and gastrointestinal tracts are directly connected and both oral and gut microbiota have been demonstrated to affect the development of systemic diseases (Uchiyama et al., 2019), the interaction between the two microbiotas is meaningful to be explored. Clarification of the exact contribution of specific gut microbe taxa to periodontitis can bring new opportunities for more efficient prevention and control of periodontitis. Although efforts have already been made to elucidate the association between periodontitis and gut microbiota, no evidence for the causal effect was proposed. Furthermore, even though studies have found that periodontitis patients have a phenotype of dysbiosis of the gut microbiota, it’s the result of a multifactorial combination and the strain-specific changes of diverse microbiota taxa are inconsistent. In addition, the composition of the gut microbiota may vary due to inconsistencies in the staging of periodontitis, gender ratio, and ethnicity of the populations in different studies. The above factors hinder the inference of a specific causal effect on the risk of periodontitis and gut microbiota taxa.

As aforementioned, MR is a perfect study design to elucidate the causal effect between potential risk factors and diseases of interest. Recently, many MR studies have been applied to clarify modest risk factors for periodontitis. In these studies, the risk of periodontitis can be exaggerated by tobacco, alcohol, and genetically proxied obesity (Baumeister et al., 2021; Dong et al., 2022), while being suppressed by high micronutrients, fiber, and omega-3 fatty acids intake (Heo et al., 2022; Watson et al., 2022). Through exploring the factors that modulate the risk of periodontitis, MR studies facilitate the recommendation of public health policies and clinical interventions that effectively reduce the incidence and social burden of periodontitis. Meanwhile, there are also some factors that were linked to periodontitis in previous epidemiological observational studies but are demonstrated to have no causal association with periodontitis via MR studies, such as arthritis and psoriasis (Baurecht et al., 2022; Yin et al., 2022), which are not recommended as a target for the prevention and control of periodontitis. Compared with previous MR studies, our study is more comprehensive, revealing the causal effect of 196 gut microbiome taxa on periodontitis while previous MR studies only focus on less than 10 exposures of interest.

So far, this work is the pioneer MR study using large-scale gut microbiome and periodontitis genetic data to explore whether gut microbiota is causally related to the risk of periodontitis. The prominent advantage of our study is that the execution of the MR method robustly diminishes the interference of reverse causal associations and confounding factors. What’s more, our MR study encompasses the widest range of the population at a minimal cost, which may be more practical and convincing than conventional observational studies. However, some limitations should be noted. First, since participants in the GWAS are predominantly of European ancestry, extrapolation of our findings to other ethnic groups may be constrained. Second, given the biological plausibility and sophisticated pathobiology of periodontitis as well as the polyphasic process of statistics, the application of a strict multiple-testing correction may be so conservative that partially potential strains that are causally correlated to periodontitis are overlooked. Therefore, we didn’t implement multiple correlations. Third, since our study aimed to elucidate the risk factors for periodontitis to achieve comprehensive clinical intervention and reduce the incidence, we target the unidirectional role of 196 gut microbiota taxa on periodontitis. Fourth, the exact mechanisms by which the as-mentioned gut microbiota taxa influence the risk of periodontitis haven’t been totally investigated in this study.

## Conclusions

In summary, this study innovatively demonstrates the causal relationship between gut microbiota and periodontitis through MR analyses and reveals the impact of specific gut microbiota taxa on the risk of periodontitis, thus providing new directions for the clinical intervention of periodontitis.

## Data availability statement

The original contributions presented in the study are included in the article/**Supplementary Material**. Further inquiries can be directed to the corresponding authors.

## Ethics statement

The studies involving human participants were reviewed and approved by Hospital of Stomatology, Guanghua School of Stomatology, Guangdong Provincial Key Laboratory of Stomatology, Sun Yat-sen University. Written informed consent for participation was not required for this study in accordance with the national legislation and the institutional requirements.

## Author contributions

SL, YL, and SW were responsible for the study conception and design. SL, WL, and QL participated in data analysis and interpretation. SL, MZ, and XW participated in figure and table drawing. SL and SW completed the manuscript writing and revision. All authors contributed to the article and approved the submitted version.

## References

[B1] Abusleme L. Hoare A. Hong B. Y. Diaz P. I. (2021). Microbial signatures of health, gingivitis, and periodontitis. Periodontol 2000. 86 (1), 57–78. doi: 10.1111/prd.12362 33690899

[B2] Bailen M. Bressa C. Martinez-Lopez S. Gonzalez-Soltero R. Lominchar M. G. M. San Juan C.. (2020). Microbiota features associated with a high-Fat/Low-Fiber diet in healthy adults. Front. Nutr. 7, 583608. doi: 10.3389/fnut.2020.583608 33392236 PMC7775391

[B3] Barbe A. G. Javadian S. Rott T. Scharfenberg I. Deutscher H. C. D. Noack M. J.. (2020). Objective masticatory efficiency and subjective quality of masticatory function among patients with periodontal disease. J. Clin. Periodontol. 47 (11), 1344–1353. doi: 10.1111/jcpe.13364 32892357

[B4] Bartold P. M. (2018). Lifestyle and periodontitis: the emergence of personalized periodontics. Periodontol 2000. 78 (1), 7–11. doi: 10.1111/prd.12237 30198129

[B5] Baumeister S. E. Freuer D. Nolde M. Kocher T. Baurecht H. Khazaei Y.. (2021). Testing the association between tobacco smoking, alcohol consumption, and risk of periodontitis: a mendelian randomization study. J. Clin. Periodontol. 48 (11), 1414–1420. doi: 10.1111/jcpe.13544 34472130

[B6] Baurecht H. Freuer D. Welker C. Tsoi L. C. Elder J. T. Ehmke B.. (2022). Relationship between periodontitis and psoriasis: a two-sample mendelian randomization study. J. Clin. Periodontol. 49 (6), 573–579. doi: 10.1111/jcpe.13620 35362630 PMC9117489

[B7] Billings M. Holtfreter B. Papapanou P. N. Mitnik G. L. Kocher T. Dye B. A. (2018). Age-dependent distribution of periodontitis in two countries: findings from NHANES 2009 to 2014 and SHIP-TREND 2008 to 2012. J. Clin. Periodontol. 45, S130–SS48. doi: 10.1111/jcpe.12944 29926501

[B8] Bjorkhaug S. T. Aanes H. Neupane S. P. Bramness J. G. Malvik S. Henriksen C.. (2019). Characterization of gut microbiota composition and functions in patients with chronic alcohol overconsumption. Gut Microbes 10 (6), 663–675. doi: 10.1080/19490976.2019.1580097 30894059 PMC6866679

[B9] Bowden J. Smith G. D. Burgess S. (2015). Mendelian randomization with invalid instruments: effect estimation and bias detection through egger regression. Int. J. Epidemiol 44 (2), 512–525. doi: 10.1093/ije/dyv080 26050253 PMC4469799

[B10] Bowden J. Smith G. D. Haycock P. C. Burgess S. (2016). Consistent estimation in mendelian randomization with some invalid instruments using a weighted median estimator. Genet. Epidemiol 40 (4), 304–314. doi: 10.1002/gepi.21965 27061298 PMC4849733

[B11] Burgess S. Thompson S. G. Collaboration CCG (2011). Avoiding bias from weak instruments in mendelian randomization studies. Int. J. Epidemiol 40 (3), 755–764. doi: 10.1093/ije/dyr036 21414999

[B12] Chapple I. L. C. Genco R. AAP WGJE (2013). Diabetes and periodontal diseases: consensus report of the joint EFP/AAP workshop on periodontitis and systemic diseases. J. Clin. Periodontol. 40, S106–SS12. doi: 10.1902/jop.2013.1340011 23627322

[B13] Chen X. Hong X. S. Gao W. J. Luo S. L. Cai J. H. Liu G. C.. (2022). Causal relationship between physical activity, leisure sedentary behaviors and COVID-19 risk: a mendelian randomization study. J. Transl. Med. 20 (1), 216. doi: 10.1186/s12967-022-03407-6 35562752 PMC9100292

[B14] Chen X. Kong J. Q. Pan J. X. Huang K. Zhou W. H. Diao X. Y.. (2021). Kidney damage causally affects the brain cortical structure: a mendelian randomization study. Ebiomedicine 72, 103592. doi: 10.1016/j.ebiom.2021.103592 34619639 PMC8498227

[B15] Chen M. Xie C. R. Shi Y. Z. Tang T. C. Zheng H. (2022). Gut microbiota and major depressive disorder: a bidirectional mendelian randomization. J. Affect. Disord. 316, 187–193. doi: 10.1016/j.jad.2022.08.012 35961601

[B16] Christensen H. Bosse J. Angen O. Norskov-Lauritsen N. Bisgaard M. (2020). Immunological and molecular techniques used for determination of serotypes in pasteurellaceae. Method Microbiol. 47, 117–149. doi: 10.1016/bs.mim.2020.01.002

[B17] Devriese S. Eeckhaut V. Geirnaert A. Van den Bossche L. Hindryckx P. Van de Wiele T.. (2017). Reduced mucosa-associated butyricicoccus activity in patients with ulcerative colitis correlates with aberrant claudin-1 expression. J. Crohns Colitis 11 (2), 229–236. doi: 10.1093/ecco-jcc/jjw142 27484096

[B18] Dong J. Y. Gong Y. X. Chu T. D. Wu L. X. Li S. S. Deng H.. (2022). Mendelian randomization highlights the causal association of obesity with periodontal diseases. J. Clin. Periodontol. 49 (7), 662–671. doi: 10.1111/jcpe.13640 35569024

[B19] Eckburg P. B. Bik E. M. Bernstein C. N. Purdom E. Dethlefsen L. Sargent M.. (2005). Diversity of the human intestinal microbial flora. Science 308 (5728), 1635–1638. doi: 10.1126/science.1110591 15831718 PMC1395357

[B20] Emdin C. A. Khera A. V. Kathiresan S. (2017). Mendelian randomization. JAMA 318 (19), 1925–1926. doi: 10.1001/jama.2017.17219 29164242

[B21] Genco R. J. Sanz M. (2020). Clinical and public health implications of periodontal and systemic diseases: an overview. Periodontol 2000. 83 (1), 7–13. doi: 10.1111/prd.12344 32385880

[B22] Hajishengallis G. Chavakis T. Lambris J. D. (2020). Current understanding of periodontal disease pathogenesis and targets for host-modulation therapy. Periodontol 2000. 84 (1), 14–34. doi: 10.1111/prd.12331 32844416 PMC7457922

[B23] Hajishengallis G. Liang S. Payne M. A. Hashim A. Jotwani R. Eskan M. A.. (2011). Low-abundance biofilm species orchestrates inflammatory periodontal disease through the commensal microbiota and complement. Cell Host Microbe 10 (5), 497–506. doi: 10.1016/j.chom.2011.10.006 22036469 PMC3221781

[B24] Heo H. Bae J. H. Amano A. Park T. Choi Y. H. (2022). Supplemental or dietary intake of omega-3 fatty acids for the treatment of periodontitis: a meta-analysis. J. Clin. Periodontol. 49 (4), 362–377. doi: 10.1111/jcpe.13603 35141945

[B25] Higbee D. H. Granell R. Sanderson E. Davey Smith G. Dodd J. W. (2021). Lung function and cardiovascular disease: a two-sample mendelian randomisation study. Eur. Respir. J. 58 (3), 2003196. doi: 10.1183/13993003.03196-2020 33574079

[B26] Hu Z. Zhou F. X. Xu H. L. (2022). Circulating vitamin c and d concentrations and risk of dental caries and periodontitis: a mendelian randomization study. J. Clin. Periodontol. 49 (4), 335–344. doi: 10.1111/jcpe.13598 35112385

[B27] Janakiram C. Dye B. A. (2020). A public health approach for prevention of periodontal disease. Periodontol 2000. 84 (1), 202–214. doi: 10.1111/prd.12337 32844412 PMC7457923

[B28] Jia X. Jia L. Mo L. Yuan S. Zheng X. He J.. (2019). Berberine ameliorates periodontal bone loss by regulating gut microbiota. J. Dent. Res. 98 (1), 107–116. doi: 10.1177/0022034518797275 30199654

[B29] Kamat M. A. Blackshaw J. A. Young R. Surendran P. Burgess S. Danesh J.. (2019). PhenoScanner V2: an expanded tool for searching human genotype-phenotype associations. Bioinformatics 35 (22), 4851–4853. doi: 10.1093/bioinformatics/btz469 31233103 PMC6853652

[B30] Kelly N. El Karim I. (2020). Periodontitis may be associated with respiratory diseases such as asthma, copd, and pneumonia. J. Evid-Based Dent. Pr. 20 (4), 101498. doi: 10.1016/j.jebdp.2020.101498 33303090

[B31] Kurilshikov A. Medina-Gomez C. Bacigalupe R. Radjabzadeh D. Wang J. Demirkan A.. (2021). Large-Scale association analyses identify host factors influencing human gut microbiome composition. Nat. Genet. 53 (2), 156–165. doi: 10.1038/s41588-020-00763-1 33462485 PMC8515199

[B32] Li L. Bao J. Chang Y. Wang M. Chen B. Yan F. (2021). Gut microbiota may mediate the influence of periodontitis on prediabetes. J. Dent. Res. 100 (12), 1387–1396. doi: 10.1177/00220345211009449 33899584

[B33] Ni J. J. Xu Q. Yan S. S. Han B. X. Zhang H. Wei X. T.. (2022). Gut microbiota and psychiatric disorders: a two-sample mendelian randomization study. Front. Microbiol. 12, 4178. doi: 10.3389/fmicb.2021.737197 PMC8856606 35185808

[B34] Ormerod K. L. Wood D. L. A. Lachner N. Gellatly S. L. Daly J. N. Parsons J. D.. (2016). Genomic characterization of the uncultured bacteroidales family S24-7 inhabiting the guts of homeothermic animals. Microbiome 4, 36. doi: 10.1186/s40168-016-0181-2 27388460 PMC4936053

[B35] Page R. C. Eke P. I. (2007). Case definitions for use in population - based surveillance of periodontitis. J. Periodontol. 78 (7), 1387–1399. doi: 10.1902/jop.2007.060264 17608611

[B36] Papapanou P. N. Sanz M. Buduneli N. Dietrich T. Feres M. Fine D. H.. (2018). Periodontitis: consensus report of workgroup 2 of the 2017 world workshop on the classification of periodontal and peri-implant diseases and conditions. J. Periodontol. 89, S173–SS82. doi: 10.1002/JPER.17-0721 29926951

[B37] Purushe J. Fouts D. E. Morrison M. White B. A. Mackie R. I. Coutinho P. M.. (2010). Comparative genome analysis of prevotella ruminicola and prevotella bryantii: insights into their environmental niche. Microb. Ecol. 60 (4), 721–729. doi: 10.1007/s00248-010-9692-8 20585943

[B38] Sanna S. van Zuydam N. R. Mahajan A. Kurilshikov A. Vila A. V. Vosa U.. (2019). Causal relationships among the gut microbiome, short-chain fatty acids and metabolic diseases. Nat. Genet. 51 (4), 600–605. doi: 10.1038/s41588-019-0350-x 30778224 PMC6441384

[B39] Shungin D. Hawort S. Divaris K. Agler C. S. Kamatani Y. Lee M. K.. (2019). Genome-wide analysis of dental caries and periodontitis combining clinical and self-reported data. Nat. Commun. 10, 2773. doi: 10.1038/s41467-019-10630-1 31235808 PMC6591304

[B40] Staley J. R. Blackshaw J. Kamat M. A. Ellis S. Surendran P. Sun B. B.. (2016). PhenoScanner: a database of human genotype-phenotype associations. Bioinformatics 32 (20), 3207–3209. doi: 10.1093/bioinformatics/btw373 27318201 PMC5048068

[B41] Tonetti M. S. Van Dyke T. E. Working group 1 of the joint EFPAAPw (2013). Periodontitis and atherosclerotic cardiovascular disease: consensus report of the joint EFP/AAP workshop on periodontitis and systemic diseases. J. Clin. Periodontol. 40 (Suppl 14), S24–S29. doi: 10.1111/jcpe.12089 23627332

[B42] Uchiyama K. Naito Y. Takagi T. (2019). Intestinal microbiome as a novel therapeutic target for local and systemic inflammation. Pharmacol. Therapeut 199, 164–172. doi: 10.1016/j.pharmthera.2019.03.006 30877020

[B43] Veller C. Kleckner N. Nowak M. A. (2019). A rigorous measure of genome-wide genetic shuffling that takes into account crossover positions and mendel’s second law. Proc. Natl. Acad. Sci. U.S.A. 116 (5), 1659–1668. doi: 10.1073/pnas.1817482116 30635424 PMC6358705

[B44] Wang Y. Y. Sakka M. Yagi H. Kaneko S. Katsuzaki H. Kunitake E.. (2018). Ruminiclostridium josui Abf62A-Axe6A: a tri-functional xylanolytic enzyme exhibiting alpha-l-arabinofuranosidase, endoxylanase, and acetylxylan esterase activities. Enzyme Microb. Tech 117, 1–8. doi: 10.1016/j.enzmictec.2018.05.016 30037546

[B45] Watson S. Woodside J. V. Winning L. Wright D. M. Srinivasan M. McKenna G. (2022). Associations between self-reported periodontal disease and nutrient intakes and nutrient-based dietary patterns in the UK biobank. J. Clin. Periodontol. 49 (5), 428–438. doi: 10.1111/jcpe.13604 35170067 PMC9315140

[B46] Xiao S. W. Liu C. Chen M. J. Zou J. F. Zhang Z. M. Cui X.. (2020). Scutellariae radix and coptidis rhizoma ameliorate glycolipid metabolism of type 2 diabetic rats by modulating gut microbiota and its metabolites. Appl. Microbiol. Biot 104 (1), 303–317. doi: 10.1007/s00253-019-10174-w 31758238

[B47] Xu Q. Ni J. J. Han B. X. Yan S. S. Wei X. T. Feng G. J.. (2022). Causal relationship between gut microbiota and autoimmune diseases: a two-sample mendelian randomization study. Front. Immunol. 12. doi: 10.3389/fimmu.2021.746998 PMC8819003 35140703

[B48] Yan J. Herzog J. W. Tsang K. Brennan C. A. Bower M. A. Garrett W. S.. (2016). Gut microbiota induce IGF-1 and promote bone formation and growth. P Natl. Acad. Sci. U.S.A. 113 (47), E7554–E7E63. doi: 10.1073/pnas.1607235113 PMC5127374 27821775

[B49] Yavorska O. O. Burgess S. (2017). MendelianRandomization: an r package for performing mendelian randomization analyses using summarized data. Int. J. Epidemiol 46 (6), 1734–1739. doi: 10.1093/ije/dyx034 28398548 PMC5510723

[B50] Yin K. J. Huang J. X. Wang P. Yang X. K. Tao S. S. Li H. M.. (2022). No genetic causal association between periodontitis and arthritis: a bidirectional two-sample mendelian randomization analysis. Front. Immunol. 13, 808832. doi: 10.3389/fimmu.2022.808832 35154127 PMC8825874

[B51] Zhou H. Liu J. Zhang Y. Fang W. Yang Y. Hong S.. (2019). Gut microbiota and lung cancer: a mendelian randomisation study. J. Thorac. Oncol. 14 (10), S510–S5S1. doi: 10.1016/j.jtocrr.2020.100042

[B52] Zhu Y. Li Y. Liu M. Hu X. D. Zhu H. Q. (2020). Guizhi fuling wan, Chinese herbal medicine, ameliorates insulin sensitivity in PCOS model rats with insulin resistance via remodeling intestinal homeostasis. Front. Endocrinol. 11, 575. doi: 10.3389/fendo.2020.00575 PMC7482315 32973686

