# Supplementary material for: Causal effects of gut microbiota on the risk of periodontitis: a two-sample Mendelian randomization study
Source: Front Cell Infect Microbiol. 2023 May 25;13:1160993. doi: 10.3389/fcimb.2023.1160993 (PMC10248501; doi:10.3389/fcimb.2023.1160993)
Supplement: Supplementary file 1 [file Table_1.docx]

Supplementary Material

**Causal Effects of Gut Microbiota on the Risk of Periodontitis: A Two-sample Mendelian Randomization Study**

Shulu Luo, **Shuyi Wu** *** & Yan Li ***

*** Correspondence:**

Corresponding Author

Dr. Shuyi Wu

E-mail: wushuyi@mail.sysu.edu.cn

Hospital of Stomatology, Guanghua School of Stomatology, Guangdong Provincial Key Laboratory of Stomatology, Sun Yat-sen University, Guangzhou 510055, P. R. China.

Prof. Yan Li

E-mail: liy8@mail.sysu.edu.cn

Hospital of Stomatology, Guanghua School of Stomatology, Guangdong Provincial Key Laboratory of Stomatology, Sun Yat-sen University, Guangzhou 510055, P. R. China.

## Supplementary Figures


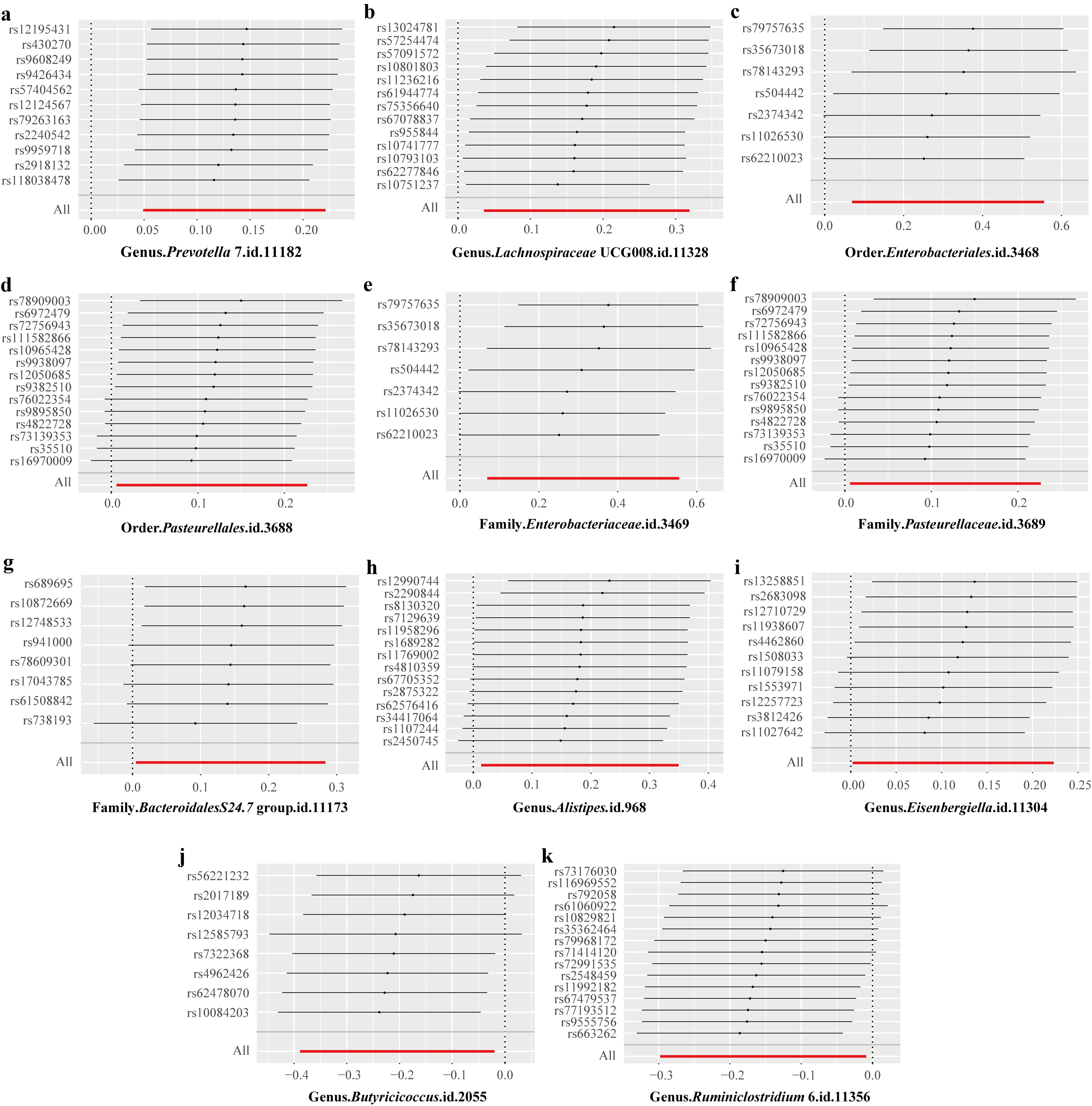


**Supplementary Figure 1.** **Leave-one-out analyses for the causal estimates of 11 gut microbiota taxa on the risk of periodontitis.** (MR, Mendelian randomization)


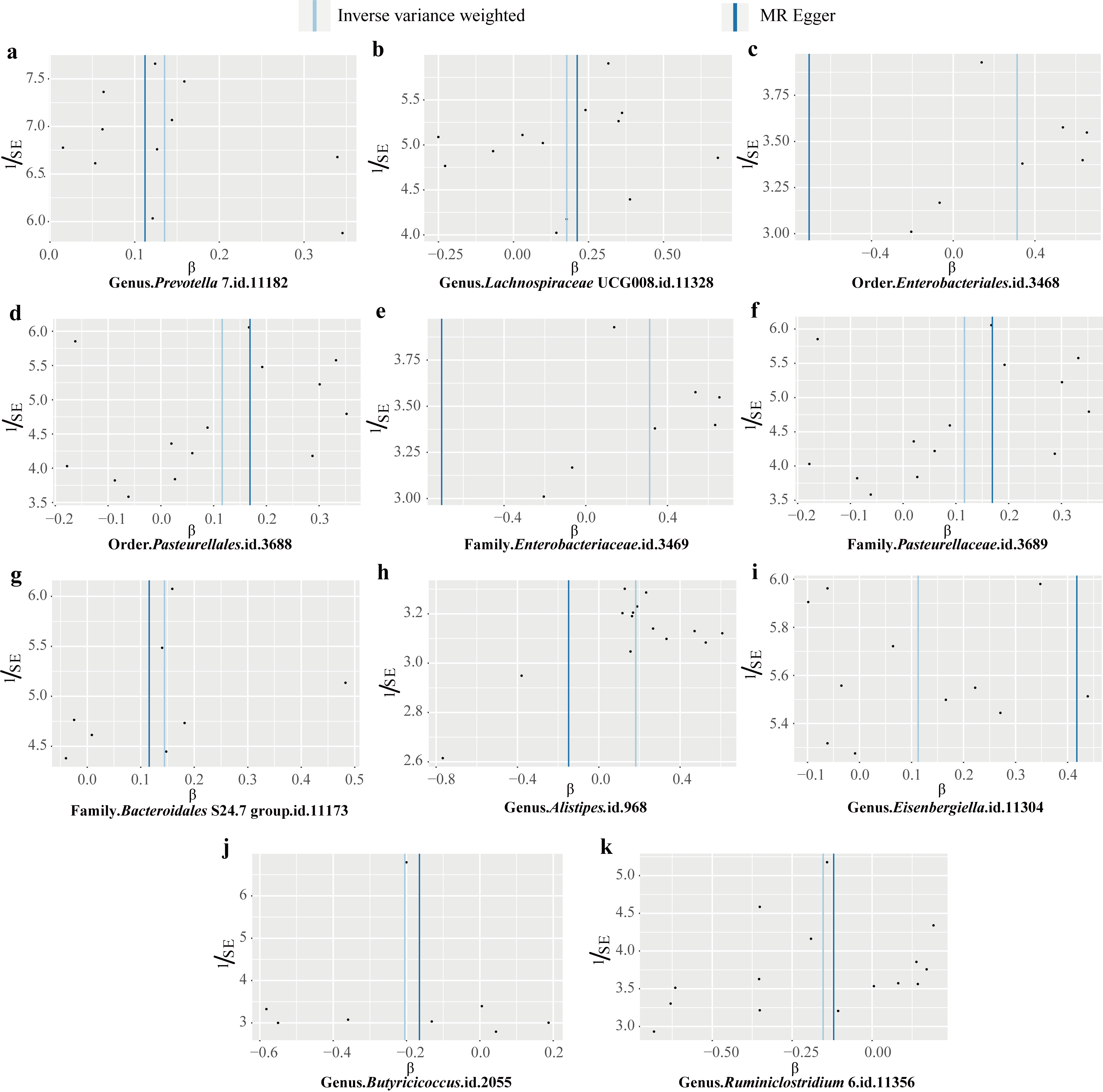


**Supplementary Figure 2.** **Funnel plots from 11 gut microbiota taxa on the risk of periodontitis.** (MR, mendelian randomization)
